# Polymerase iota (POLI) confers radioresistance of esophageal squamous cell carcinoma by regulating RAD51 stability and facilitating homologous recombination

**DOI:** 10.1038/s41420-023-01541-8

**Published:** 2023-08-09

**Authors:** Xiaoqing Li, Dexuan Gao, Fei Shen, Hengrui Chen, Zhuqiang Zhang, Chao He, Aidi Gao, Yue Lang, Xiaozhong Zhu, Jundong Zhou, Zeng-Fu Shang, Wei-Qun Ding, Ji Zhu

**Affiliations:** 1 Suzhou Cancer Center Core Laboratory, Nanjing Medical University Affiliated Suzhou Hospital, Suzhou, Jiangsu China; 2 Department of Urology, Shandong Provincial Hospital Affiliated to Shandong First Medical University, Jinan, Shandong China; 3 Jiangsu Institute of Hematology, National Clinical Research Center for Hematologic Diseases, NHC Key Laboratory of Thrombosis and Hemostasis, The First Affiliated Hospital of Soochow University, Suzhou, China; 4 Department of Radiation Oncology, Nanjing Medical University Affiliated Suzhou Hospital, Suzhou, Jiangsu China; 5 Department of Radiation Medicine, Baotou Medical College, Baotou, China; 6 State Key Laboratory of Radiation Medicine and Protection, School of Radiation Medicine and Protection, Medical College of Soochow University, Collaborative Innovation Center of Radiation Medicine of Jiangsu Higher Education Institutions, Soochow University, Suzhou, China; 7 Department of Cardiothoracic Surgery, Affiliated Hospital of Jiangsu University, Zhenjiang, Jiangsu China; 8 Department of Pathology, University of Oklahoma Health Science Center, Oklahoma City, OK USA; 9 Zhejiang Cancer Hospital, Hangzhou Institute of Medicine (HIM), Chinese Academy of Sciences, Hangzhou, Zhejiang, 310022 China

**Keywords:** Oesophageal cancer, Cancer therapeutic resistance

## Abstract

Radiotherapy resistance is an important and urgent challenge in the clinical management of esophageal squamous carcinoma (ESCC). However, the factors mediating the ESCC resistance to radiotherapy and its underlying molecular mechanisms are not fully clarified. Our previous studies have demonstrated the critical role of DNA polymerase iota (POLI) in ESCC development and progression, here, we aimed to investigate the involvement of POLI in ESCC radiotherapy resistance and elucidate the underlying molecular mechanism. We found that highly expressed POLI was correlated with shorter overall survival of ESCC patients received radiotherapy. Down-regulation of POLI sensitized ESCC to IR, prolonged γH2AX foci in nuclei and comet tails after IR. HR but not NHEJ repair is inhibited in POLI-deficient ESCC cells. POLI stabilizes RAD51 protein via competitively binding with and blocking the interaction between RAD51 and E3 ligase XIAP and XIAP-mediated ubiquitination. Furthermore, loss of POLI leads to the activation of GAS signaling. Our findings provide novel insight into the role of POLI in the development of radioresistance mediated by stabilizing RAD51 protein in ESCC.

## Introduction

Esophageal Cancer (EC) is a commonly occurring malignancy ranking sixth in global mortality rates due to cancer-related deaths [1]. ESCC (esophageal squamous cell carcinoma) is among the most frequent histological type of EC in East Asia while contributing to almost 90% or more of all EC cases [2]. Radiationtherapy alone or in combination with chemotherapy or immunotherapy is the standard treatment for unresectable or other ESCC. A significant percentage of ESCC patients who undergo radiation therapy (RT) experience recurrence after the treatment, which has been correlated with metastasis and a poor prognosis [3]. As a result, it’s of great significance to understand the molecular mechanisms underlying the radioresistance among patients with ESCC.

DNA is the principal cellular target for the biological effects of ionizing radiation (IR). It is DNA double-strand breaks (DSBs) that are the most severe and lethal types of DNA lesions caused by IR since even one DSB alone can trigger cell death or arrest the cell cycle [4]. Homologous recombination (HR) and nonhomologous end-joining (NHEJ) are the two major pathways through which DNA DSBs are repaired in higher eukaryotes. Unlike NHEJ, which is error-prone as DNA ends are damaged during processing, HR solves the problem by synthesizing homologous DNA based on template DNA. During HR repair, DNA ends are resected, generating a long 3′ single-stranded DNA tail. As soon as replication protein A (RPA) binds to single-stranded DNA(ssDNA), RAD51 replaces it, whereas Recombinase RAD51 is an ATP-dependent enzyme that catalyzes homology search and DNA strand exchange [5]. It has been shown that increased levels of RAD51 in irradiated human tumor tissues correlate with increased resistance to chemoradiotherapy and poor prognosis in a variety of human cancers, including ESCC [6, 7]. Yet, the role of RAD51 and other signal pathways in mediating HR repair and enhancing radioresistance in ESCC cells remains unclear.

A cell can replicate and bypass damaged DNA in the template strand by using TLS (Translesion DNA Synthesis). The error-prone nature of TLS is attributed to a group of DNA polymerases, many belonging to the Y-family, such as POLI, POLK, POLH, and REV1 [8]. A study revealed that POLI has the lowest fidelity to lesions across various DNA types, hence causing mutations as well as genomic instability [9]. We previously found the upregulation of POLI among ESCC tissues where POLI dysregulation drives ESCC cell proliferation by promoting cyclin D1 expression [10]. Additionally, we found that POLI significantly enhanced the invasiveness and metastasis of ESCC cells [11]. Based on clinical sample analysis, it has been shown that POLI expression in tumor tissues is closely related to lymph node metastasis in ESCC tumors [11]. In addition to this line of research on POLI’s role in ESCC progression, the present study determined that the expression levels of ESCC POLI are inversely associated with overall survival. Radiosensitivity for ESCC cells was markedly increased by depletion of endogenous POLI. In addition, we demonstrated that HR is inhibited in POLI-deficient ESCC cells mainly due to POLI stabilizes the RAD51 protein by inhibiting its interaction with its novel E3 ligase X-linked inhibitor of apoptosis (XIAP) and its ubiquitination by XIAP. A new understanding of the mechanisms behind the development of radioresistance among patients with ESCC is provided by these findings.

## Results

### Radiotherapy resistance and poor patient survival linkage to high expression of POLI in ESCC

**Fig. 1:**
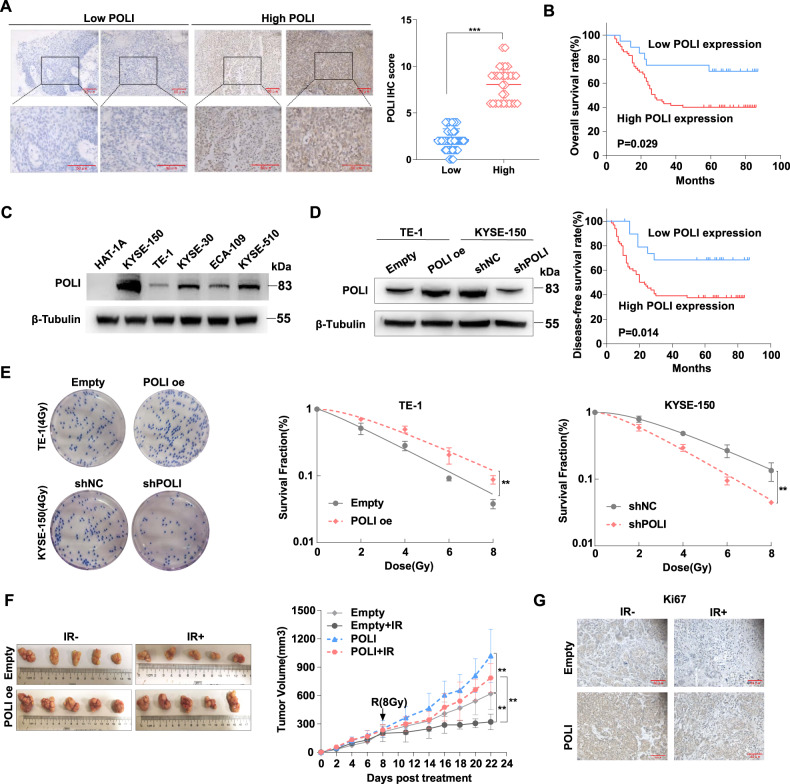
The POLI treatment enhanced the radioresistance of esophageal squamous cell carcinoma cells in vitro and in vivo. **A** ESCC patients were classified into low- and high-POLI expression groups according to IHC scores (IHC score 6 was used as a cutoff value for POLI) in all cases. Scale bar, 50 μm. *P* value was measured by Student’s *t* test. **B** Results recorded from Kaplan-Meier survival analysis for overall survival (OS) and disease-free survival (DFS) revealed significantly shortened OS (*P* = 0.029) and DFS (*P* = 0.014) for patients with high POLI expression compared to patients with low POLI expression. **C** The protein levels of POLI in ESCC and normal cell lines were detected by western blot. **D** Analysis of POLI expression in the ectopic POLI (TE-1-POLI oe vs TE-1-empty) and specific endogenous POLI knockdown (KYSE-150-shPOLI vs KYSE-150-shNC) ESCC cells using Western blot. **E** Clonogenic survival curves were generated for TE-1 and KYSE-150 cells that were exposed to 2, 4, 6, and 8 Gy X-ray irradiation. The single hit multiple target radiobiological model was used to fit the survival curves. **F** Images as well as growth curve of the volume out of ESCC tumor xenografts in nude mice. The tumor volumes were presented as the mean ± SEM of three independent experiments. *P* value was measured by Student’s *t* test. **G** The expression level of Ki67 in tumors from the mice was detected by IHC staining. ***P < 0.00, **P < 0.01.

We previously reported that the expression of POLI was upregulated in ESCC and correlated with lymph node metastasis in human ESCC [11]. We examined the expression of POLI in 85 radiotherapy patient tissues to determine whether POLI is implicated in the response of ESCC to radiotherapy by using immunohistochemistry (IHC). Based on the IHC score (Score 6 as a cutoff value) of POLI in the ESCC sores of all patients, POLI expression was divided into low-expression and high-expression groups (Fig. 1A). In Table 1, we list the clinicopathologic characteristics of patients from two groups. According to Kaplan-Meier survival analysis among patients with high and low POLI expression, OS (*P* = 0.029) and DFS (*P* = 0.014) were significantly shorter among patients with high POLI expression than those with low POL1 expression (Fig. 1B). These results suggest that expression of POLI protein is positively linked to radioresistance as well as poor prognosis of ESCC patients after radiotherapy.

**Table 1:** The clinicopathologic characteristics of eighty-five patients with esophageal cancer who received postoperative radiotherapy is presented.

Characteristics	Low expression of Polι (*n* = 20)	High expression of Polι (*n* = 65)
**Gender**		
Male	19	51
Female	1	14
**Age(year)**		
≤60	15	41
>60	5	24
**T stage**		
T1	0	2
T2	5	13
T3	15	50
**N stage**		
N0	13	28
N1	2	27
N2	4	6
N3	1	4
**TNM stage**		
IIA ~ IIB	13	39
IIIA ~ IIIC	7	26
**Pathological grade**		
G1 ~ G2	11	31
G3 ~ G4	9	34

### POLI enhances esophageal squamous cell carcinoma cell radioresistance in vitro and in vivo

To determine whether POLI involves in the radioresistance of ESCC cells, the protein levels of POLI in ESCC and normal esophageal cell lines were evaluated by western blot. As shown in Fig. 1C, POLI expression is up-regulated in ESCC cells, while KYSE-150 cells had the highest level and TE-1 had relative low level of POLI protein. Next, the POLI was knocked down in KYSE-150 cells with shRNA, and exogenously expressed in TE-1 cells (Fig. 1D). Then, ESCC cells were exposed to X-ray irradiation and given either ectopic expression of POLI (i.e. TE-1 POLI oe vs TE-1 NC) or specific knockdown endogenous POLI (i.e. KYSE-150-shPOLI vs KYSE-150-shNC). Following irradiation, POLI-deficient significantly impaired the clonogenic formation capacity, while POLI over-expression resulted in enhanced colony formation ability (Fig. 1E). Next, we determined if POLI also affects ESCC cell responses to IR in vivo. As depicted in Fig. 1F, the mice were treated IR with an 8 Gy dose (with 2 Gy/min, dose rate) when the volume of tumors reached 200mm^3^. Xenograft tumor growth was found to be dramatically delayed for POLI-deficient cells, but promoted for POLI-overexpressed cells compared to the control group after being exposed to the same IR treatment. Meanwhile, the results of IHC staining showed that POLI over-expressed ESCC cells derived tumors had higher Ki67 protein levels compared with control (Fig. 1G). Based on these results, POLI appears to increase the radioresistance of ESCC cells in in vitro and in vivo.

### POLI increases DNA DSBs repair capacity in esophageal squamous cell carcinoma cell via facilitating homologous recombination that accompanies by an enhanced RAD51 expression

**Fig. 2:**
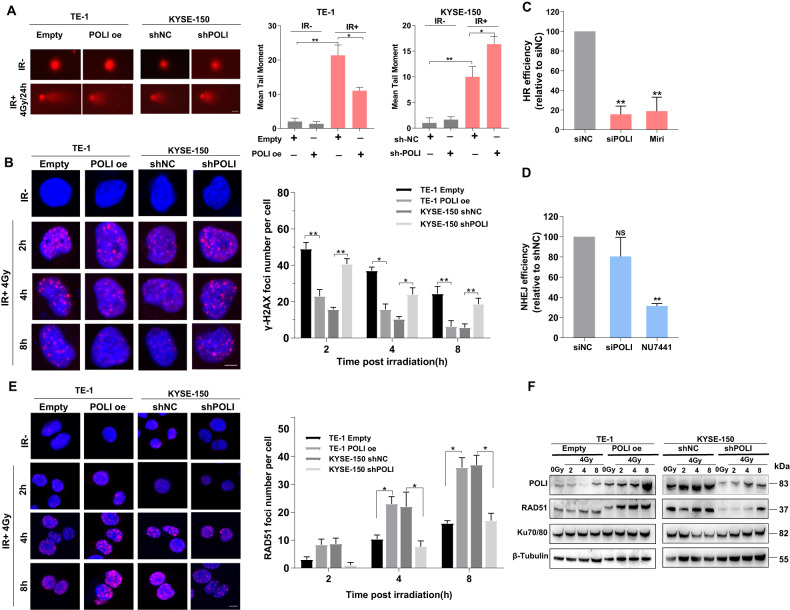
POLI facilitates the repair of DNA DSBs in esophageal squamous cell carcinoma cells via homologous recombination. **A** DNA double-strand breaks (DSBs) damage to ESCC cells treated with IR (4 Gy) was detected by using the neutral single-cell gel electrophoresis assay (comet assay). Scale bar, 20 μm. **B** Immunofluorescent staining of γ-H2AX foci was adopted at different time points after 4 Gy IR and a measured number of foci per cell. Scale bar, 5 μm. **C**, **D** CRISPR/Cas9-induced oligodeoxynucleotide (ODN) detecting system was applied to monitor the efficiencies of NHEJ and HR. **E** Immunofluorescent staining of RAD51 foci was performed at several time points after 4 Gy IR and a measured number of foci per cell. Scale bar, 10μm. **F** Western blotting analysis of POLI, RAD51, and Ku70/80 expression at different times points following 4 Gy IR treatment. Data was shown as mean ± SEM. *P* value was measured by Student’s *t* test; ***P < 0.00, **P < 0.01, *P < 0.05.

To further confirm the contribution of POLI to the radioresistance of ESCC, DNA double-strand breaks (DSBs) after 4 Gy of IR were detected using a neutral single-cell gel electrophoresis assay (comet assay). At indicated time points post-IR, the comet tails of KYSE-150 shPOLI and TE-control cells are much longer than those in KYSE-150 shNC and TE-POLI oe cells (Fig. 2A). H2AX is phosphorylated on its Ser139 (γH2AX) around DNA DSBs, making it an important molecular marker of DNA damage [12]. In this study, immunofluorescence staining of γH2AX foci was applied, and POLI-deficient ESCC cells had an extended repair time when compared to ESCCs with POLI expression 4 Gy of IR (Fig. 2B). There are two main mechanisms for repairing damaged DSBs, homologous recombination (HR) and non-homologous end-joining (NHEJ). Using an oligodeoxynucleotide (ODN) detection technology induced by CRISPR/Cas9, the efficacy of NHEJ and HR is measured as previously described [13, 14]. It is found that with loss of POLI-expression, HR activity is markedly reduced among ESCC cells, but NHEJ-mediated DSB repairs were unaffected (Fig. 2C, D). RAD51 foci have become a popular method of identifying DSB-repairing cells since this protein plays a vital role in HR repair. Figure 2E shows that the IR-induced RAD51 foci were higher in the POLI-expressing ESCC cells than in the POLI-deficient cells (compared with control cells). It is quite interesting to note that immunoblotting analysis revealed that RAD51 protein levels were dramatically elevated in POLI-expressing ESCC cells compared to control cells (Fig. 2F).

### POLI interacts with XIAP and prevents the ubiquitination and degradation of RAD51

**Fig. 3:**
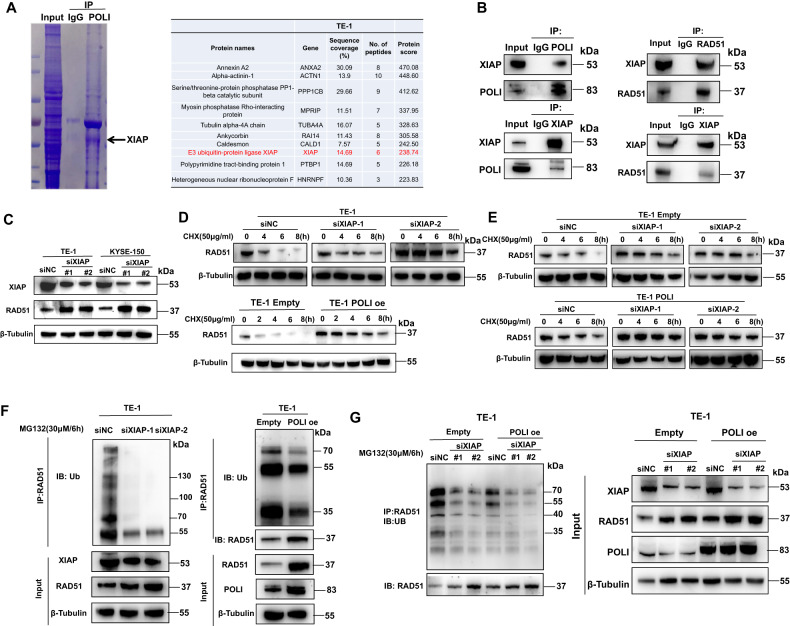
POLI interacts with XIAP and prevents the ubiquitination and degradation of RAD51. **A** The results of MS analysis suggested that POLI could interact with E3 ligase XIAP. **B** The immunoprecipitation assay was used to verify the interaction between XIAP and POLI or RAD51 using whole cell lysate from TE-1 cells. **C** Western blotting was analysis of RAD51 and XIAP expression in the specific endogenous XIAP knockdown ESCC cells. **D** The specific endogenous XIAP knockdown TE-1 cells and the POLI TE-1 cells with ectopic expression were treated with cycloheximide (CHX, 50 μg/ml) for the indicated times and western blotting was performed to detect RAD51 protein levels. **E** Western blotting was performed to detect RAD51 protein levels in down-regulating XIAP of TE-1-POLI oe and TE-1-empty cells treated with cycloheximide (CHX, 50 μg/ml). **F** Western blotting following immunoprecipitation assay was performed to detect the signal of polyubiquitinated RAD51 in the specific endogenous XIAP knockdown TE-1 cells and the POLI TE-1 cells with ectopic expression that were exposed to MG132 (30 μM) for 6 h. **G** Western blotting was performed after immunoprecipitation to detect polyubiquitinated RAD51 protein levels in down-regulating XIAP of TE-1-empty cells and TE-1-POLI-oe cells.

To better understand the role of POLI on RAD51 stability regulation, immunoprecipitation combined with mass spectrometry analysis was carried out to identify specific proteins that may interact with POLI in this process. The MS analysis indicates that POLI could interact with the E3 ligase XIAP (Fig. 3A). The XIAP protein combines with the E3 ubiquitin ligase to ubiquitinate a range of targets, including caspases, thereby conferring resistance to chemotherapy or radiotherapy mediated by apoptosis suppression in cancer cells [15]. We speculated that POLI interacts with XIAP to disrupt XIAP’s interaction with RAD51 to maintain RAD51 protein levels in ESCC cells. As a first step towards verifying this hypothesis, we determine how XIAP interacts with POLI or RAD51. ESCC cells showed an interaction between POLI and XIAP as shown in Fig. 3B. Moreover, we also observed an interaction between XIAP and RAD51 in ESCC cells (Fig. 3B), whereas the knockdown of XIAP increased RAD51 protein levels (Fig. 3C). The expression of POLI also prevented significant degradation of the RAD51 protein in ESCC cells (Fig. 3D). Similarly, down-regulating XIAP attenuated RAD51 degradation (Fig. 3D), which was further enhanced by overexpression of POLI (Fig. 3E). Moreover, the polyubiquitinated RAD51 signal was much weaker in POLI over-expressing, XIAP deficient cells (Fig. 3F) than in control cells. The over-expression of POLI in ESCC cells may also enhance the stability and inhibit the ubiquitination of RAD51 following the down-regulation of XIAP (Fig. 3E, G). These results suggest that POLI regulates RAD51 protein stability via the mechanism of altering the XIAP-mediated degradation of RAD51.

### POLI regulates ubiquitin-mediated RAD51 degradation by blocking an interaction among RAD51 and XIAP via a competitive binding manner

**Fig. 4:**
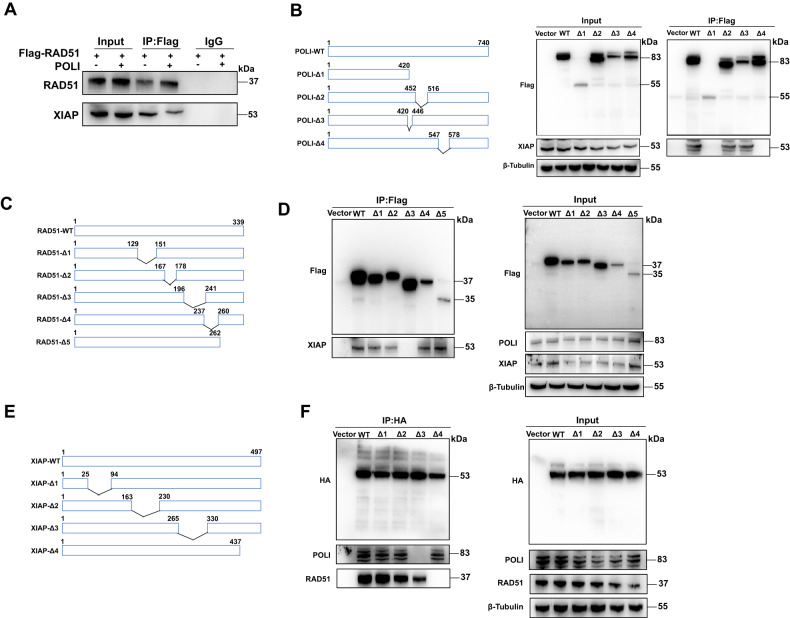
POLI inhibits ubiquitin-mediated degradation of RAD51 by blocking the interaction between RAD51 and XIAP in a competitive binding manner. **A** Over-expression of POLI impaired the interaction between XIAP and RAD51 proteins. **B** Schematic diagram of the POLI protein domains. Characterize the regions of POLI mediating its binding with XIAP. **C** Schematic diagram of RAD51 protein domains. **D** Characterize the regions of RAD51 mediating its binding with XIAP. **E** Schematic diagram of XIAP protein domains. **F** Identification of the regions of XIAP that mediate its binding with POLI and RAD51.

To confirm whether POLI and XIAP were competitively binding with RAD51, XIAP and RAD51 complexes were immunoprecipitated from POLI-expressing and POLI-deficient ESCC cells. The expression of POLI significantly impaired the interaction between RAD51 and XIAP, as shown in Fig. 4A. We further characterized the POLI regions that mediate its binding with XIAP by transfecting them with Flag-tagged truncated expression plasmids covering various domains of POLI and then analyzing them using co-IP. According to our results, POLI interacts with XIAP through its REV1-interacting region (RIR) (Fig. 4B). Further, the 196–241 amino acids of RAD51 are necessary for binding to XIAP (Fig. 4C, D). After narrowing the truncated XIAP domains further, we found that the loss of amino acids 437–487 abolishes the interaction between XIAP and RAD51 (Fig. 4E, F).

### Loss of POLI enhances IR-induced immune response in ESCC cells

**Fig. 5:**
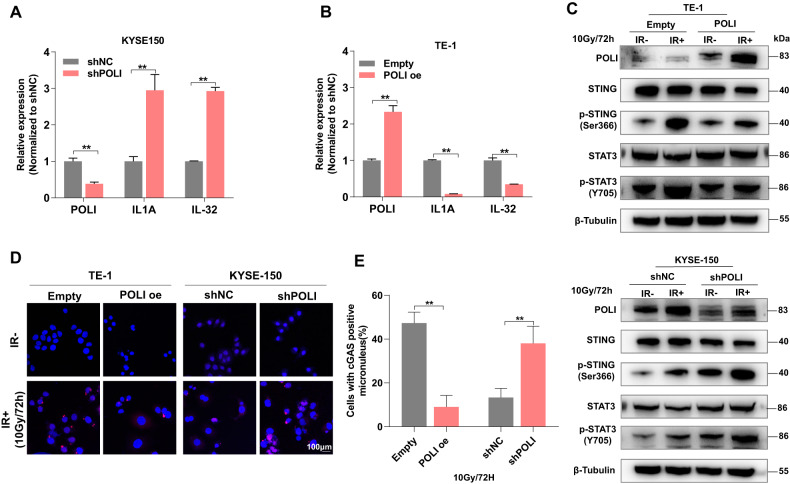
The loss of POLI enhances the IR-induced immune response in ESCC cells. **A**, **B** Quantitative RT-PCR was performed to detect IL1A and IL-32 in ESCC cells expressing either down-regulated or over-expressed POLI. (**C**) Western blotting was used to detect the expression of p-STAT3, and p-STING in eESCC cells at 72 h following 10 Gy IR treatment. **D**, **E** Immunofluorescent staining of cGAS micronuclei was adopted at 72 h after 10 Gy IR. We measured the number of cGAS-positive micronuclei per cell. Scale bar, 100μm. The data was represented as mean ± SEM. *P* value measured by paired Student’s *t* test; **P < 0.01.

In several recent studies, RAD51 exerted a protective effect against the leakage of cleaved and resected single-stranded DNA into the cytoplasm, as well as the blocking of the innate immune reaction caused by IR [16]. Our data indicated that the expression of many genes involved in innate immunity is up-regulated significantly in POLI-deficient ESCC cells as compared with POLI-proficient ESCC cells (Fig. 5A, B). Micronuclei resulting from IR are the main reason for triggering pro-inflammatory signaling cascades [17, 18]. In view of this, we examined if the suppression of POLI would exacerbate the formation of cGAS-positive micronuclei. After irradiation with 10 Gy X-ray, ESCC cells lacking POLI appearance significantly increased the amounts of micronuclei and cGAS-positive micronuclei (8.00% in TE-1-POLI cells as compared to 47.33% in TE-1 cells; 13.33% in KYSE-150-shNC cells as compared to 38.00% in KYSE-150-shPOLI cells) (Fig. 5D, E). We performed western blot analysis to determine if an increase in micronuclei activates STING. Upon irradiation, POLI-deficient cells showed stronger STING phosphorylation than POLI-expressing cells. After irradiation, STAT3 phosphorylation was significantly higher in POLI-deficient cells than in POLI-proficient cells (Fig. 5C), consistent with STAT3 being a target of the cGAS-STING signal pathway in response to cytosolic DNA. In this study, we determined that loss of POLI results in increasing micronuclei as well as increased innate immune response signaling.

## Discussion

Genome stability is ensured by TLS DNA polymerases by replicating across damaged DNA in response to replication stress. In addition, several studies showed that TLS DNA polymerases are also involved in DNA double-strand break repair. TLS across DNA lesion located on a 5’ overhang at the end of DSBs, which helps the formation of a blunt end and prevents loss of DNA sequences [19]. In addition to TLS DNA polymerases, POLH has been implicated in the HR repair. It has been shown that POLH can be loaded onto synthetic D-loops and extended D-loops during the invasion of the 3’ overhang strand into the donor homologous chromosome in the course of HR repair [20]. In our study, highly expressed POLI was associated with a poor prognosis among patients with ESCC who underwent IR treatment. We have also observed an increase in the stability of RAD51 protein and HR repair efficiency as at least partial determinants of POLI-induced radioresistance in ESCC.

RAD51 levels are remarkably regulated because RAD51 has a direct impact on DNA damage repair and cellular adaptability to genotoxic agents. It has been suggested that mRNA transcriptional regulation of RAD51 may be an important mechanism to regulate HR repair protein amounts. A recent study by Liu and his colleagues showed that proteasomal degradation of RAD51 plays a major role in the IR-induced accumulation of RAD51 [21]. They found that Lamin B1 interacts with RAD51, which maintains RAD51 protein stability and facilitates nuclear accumulation of RAD51 after IR treatment. The β1-integrin has also been implicated in HR repair via promoting RAD51 protein levels in a ubiquitin-protein ligase RING1-mediated manner. Therefore, overexpression of β1-integrin is associated with chemo- and radioresistance of tumor cells [22]. By inhibiting ubiquitination-mediated degradation of RAD51, POLI could also stabilize the RAD51 protein in this study. In addition, we identified the interaction between POLI and XIAP in ESCC cells, and we found that XIAP directly interacts with RAD51 and promotes its ubiquitination and degradation.

The E3 ligase XIAP, which is a DNA damage response protein, can suppress DNA damage-induced apoptosis by selectively binding and inhibiting caspase-3, −7 and −9 [23]. XIAP promotes proteasomal degradation of the active-form of caspase-3, but not procaspase-3. Removing the RING finger domain eliminates the activity of XIAP on caspase-3 degradation [24]. Although the increased protein levels of RAD51 correlate with HR repair activity and radioresistance of cancer cells, recent study proposed that timely removal of RAD51 from damaged DNA ends through E3 ligase RFWD3-mediated ubiquitin-proteasomal degradation can facilitate the loading of other repair members, such as MCM8, onto chromatin, which is essential for HR repair [25]. In this study, the interaction between POLI and XIAP was identified using co-immunoprecipitation-coupled mass spectrometry (IP-MS) analysis, and confirmed by CO-IP and western blotting analysis. By inhibiting ubiquitination-mediated degradation of RAD51, POLI could also stabilize the RAD51 protein in this study. In addition, we identified the interaction between POLI and XIAP in ESCC cells, and we found that XIAP directly interacts with RAD51 and promotes its ubiquitination and degradation. After mapping the interaction domains between XIAP and POLI or RAD51, we found that XIAP binds to the central residues 184-257 of RAD51 and that the BIR3 domain of XIAP is necessary for POLI binding, while the RING domain is necessary for RAD51 binding. Accordingly, POLI may maintain the RAD51 protein by inhibiting the XIAP-mediated ubiquitin-proteasomal degradation of RAD51 by competitively binding with XIAP.

The deleterious mutations of *BRCA1/2* are linked to increased risk of malignancy, including breast cancer, ovarian cancer, and ESCC [26, 27]. The mutation of *BRCA1/2* genes has also been implicated in predicting therapeutic response in various cancer types. Both BRCA1 and BRCA2 can bind to RAD51 and their direct interaction facilitates the recruitment of RAD51 to DNA lesion sites and the RAD51-mediated DNA joint formation [28, 29]. Although our study showed that loss of POLI impairs IR-induced RAD51, it is still uncertain whether POLI have influences on BRCA1/2-RAD51 interaction and the DNA damage signal transduction. A recent study has found that cytosolic self-DNA, as a consequence of genomic instability, triggers a STING-dependent immune response. As a DNA-binding protein, RAD51 protects nascent DNA from MRE11-mediated excessive degradation on the replication fork [30]. Additionally, RAD51 inhibits immune response signaling by preventing nuclear single-strand DNA fragments from leaking into the cytosol [31]. Based on our findings, we found that ESCC cells deficient in POLI with a low RAD51 expression level exhibited increased numbers of IR-induced micronuclei and enhanced cGAS-STING-related inflammatory responses. Recent studies have shown that cytosolic self-DNA resulting from targeting DNA damage repair factors increases cancer cell sensitivity to PD-1 and PD-L1 blockage treatment [32, 33]. Thus, targeting the POLI-XIAP-RAD51 pathway may make ESCC cells more susceptible to immune checkpoint inhibitors.

According to our retrospective analysis, we found that highly expressed POLI significantly correlates with poor response to IR treatment in patients with ESCC. According to our findings, POLI confers radioresistance to ESCC cells by enhancing HR repair and by stabilizing the RAD51 protein by blocking the XIAP-RAD51 interaction and XIAP-mediated ubiquitin-proteasomal degradation of RAD51. Further, the downregulation of POLI elevated the activity of the cGAS-STING-related inflammatory response in ESCC cells. Despite our finding that loss of POLI impairs IR-induced RAD51, it is unclear whether POLI affects BRCA1/2-RAD51 interactions or DNA damage signal transduction.

In summary, we identified that highly expressed POLI confers the ESCC cells IR resistance by maintaining RAD51 protein stability through blocking the XIAP-RAD51 interaction and XIAP-mediated ubiquitin-proteasomal degradation of RAD51. Our findings indicate that POLI expression might be a potential molecular marker to predict clinical outcomes for patients with ESCC treated with radiotherapy and immune checkpoint inhibitors.

## Materials and Methods

### Patient tissue samples

ESCC tissue samples were obtained from 85 patients treated with radiotherapy between 2005 and 2009 at Nanjing Medical University (NMU) Affiliated Suzhou Hospital located in Jiangsu, China. Paraffin was used to embed all tissue samples. All informed consent were obtained from the patients. Ethics approval for this study was granted by the Institutional Ethics Committee of Nanjing Medical University (number: KL901200).

### Cell culture and irradiation

We purchased ESCC human cell lines TE-1 and KYSE-150 from the Shanghai Institute of Cell Research (Shanghai, China). None of the cells had been irradiated by radiation. TE-1 cells were cultured in DMEM medium (Hyclone, USA) and KYSE-150 cells were cultured in RPMI-1640 medium (Hyclone, USA) supplemented with 10% FBS (Biological Industries, Kibbutz Beit-haemek, Israel) at 37°C in 5% CO_2_. POLI stably over-expressed KYSE-150 cells was established and kept in our laboratory as previously described.[11] To generate POLI stably knockdown cell, TE-1 cells were infected with lentivirus containing the POLI-targeted shRNA sequence and selected with 1 μg/ml puromycin (Sigma-Aldrich, USA). The knockdown efficiency was verified by qPCR and western blot. Xenografted tumors and cells were irradiated with X-ray energy at a dose rate of 2 Gy/min and a 6 MeV dose rate by a linear accelerator (Varian, Palo Alto, CA, USA).

### Immunohistochemical (IHC) analyses

All tissues were embedded in paraffin wax and cut into 5μm sections. We deparaffinized tumor tissue sections in xylene and hydrated them in ethyl alcohol in various concentrations. The sections were heated for 5 min with citrate buffer (pH value: 6.0) to retrieve the antigens. A nonspecific reaction was blocked by adding 5% BSA for 30 min. Following this, sections were incubated overnight with POLI antibody (ab185686, Abcam, UK) at 1:100 dilutions. Afterward, we added an HP-conjugated antibody for one hour and developed the color with 3-3’-diaminobenzidine. Hematoxylin counterstain was applied to the dried tissues after washing, and they were briefly immersed in an ammonia-water bath before being dehydrated and mounted in Diatex. An examination as well as scoring of the stained sections is achieved with the aid of a Leica microscope (Leica Corporation, Germany). The slides were scored blind by two pathologists. The score of staining intensity was 0, 1, 2 and 3 according to no staining, weak staining, dark staining and strong staining. The rate of staining cells was scored 0, 1, 2, 3, 4 determined by <10%, 11–25%, 26–50%, >75%. Finally, the POLI histochemistry score was defined according to the staining intensity and staining cell rate.

### Analysis of cells’ ability to form colonies (colony formation assay, CFA)

We then performed the CFA, for which the TE-1, as well as KYSE-150 cells (with 200, 1000, 2000, 4000, and 6000 cells/well), were plated separately into wells of 6-well culture plates, then 24 h later, the cells were exposed to X-ray irradiation (at 0, 2, 4, 6 or 8 Gy). Then we fixed the cells with 10% methanol and stained them with 0.05% crystal violet after 14 days. Colonies containing at least 50 cells were counted. The survival fraction for the experimental group was calculated as the fraction of colonies divided by those of the control group. In the single hit multiple target radiobiological model, survival curves were fitted with single hit multiple target radiobiological models.

### RNA extraction and quantitative RT-PCR

Total RNA was extracted from cells using RNA-easy Isolation Reagent (vazyme, China). RNA concentration was determined by NanoDrop2000 (NanoDrop, USA). RT SuperMix(vazyme, China) was used to reverse transcribe 1 μg RNA. SYBR qPCR Master Mix (vazyme, China) was used for qPCR analysis. β-actin mRNA levels were used as control. qPCR was conducted on the StepOne Plus instrument (ABI, USA). The sequence of shPOLI(5’ to 3’) is GGATCTAACAGAAATGGTTGA. The sequence of siXIAP(5’ to 3’) is GCAGGUUGUAGAUAUAUCATT and GGUCAGUACAAAGUUGAAATT. Primers for β-actin, POLI, IL1A, and IL-32 were shown as follows: POLI, forward:5’-ACAAACCGGGATTTCCTACC-3’, reverse:5’-TCACACTTC CTTTCCCTTGAA-3’; β-actin, forward:5’-AGCGAGCATCCCCCAAAGTT-3’, reverse:5’-GGGCACGAAGGCTCATCATT-3’; IL1A, forward:5’-TGGCTGTTTTCTCTCACATTGC-3’, reverse:5’-TCAAACCAGGGAGGGACAAG-3’; IL-32, forward:5’-GTCTCAGTGGAGCTGGGTCA-3’, reverse:5’-CCCCTGAGCAGAAGTAGGGA-3’. Threshold cycle (Ct) was used to define the qPCR results and 2^-ΔΔCt^ was used to calculate the relative expression.

### Tumor-xenograft experiments

We purchased twenty female BALB/c nude mice (Four-weeks old) from Shanghai SLAC Laboratory Animal Co. Ltd. (Shanghai, China) and maintained them under pathogen-free conditions with free access to standard food as well as distilled water. We randomly divided nude mice into four groups on average: TE-1 empty without IR, TE-1 POLI oe without IR, TE-1 empty with IR, and TE-1 POLI oe with IR. The cells were resuspended using PBS to a concentration of 2.0×10^7^cells/ml. We then injected the nude mice with 0.20 ml of the cell suspension subcutaneously in the right groin. The size of the tumors was measured every two days with vernier calipers. To determine the tumor volume, we used the following formula: Tumor volume (V, mm^3^) = length (mm)*width^2^ (mm^2^)/2. At a dose of 8 Gy, tumors from the TE-1 empty with IR and TE-1 POLI oe with IR groups were irradiated when their volume reached 200 mm^3^. Molecular analyses and HE staining were performed two weeks later to examine tumors for molecular signs of malignancy. The nude mouse experiments were approved by the animal ethics committee of the Nanjing Medical University, and the procedures are conducted following the guidelines of the Animal Care and Use Committee at the Nanjing Medical University.

### Immunofluorescence

Irradiation of TE-1 and KYSE-150 cells on glass slides in 24-well plates with 4 Gy X-rays was carried out. During the various time intervals, the cell fixing is done using 4% paraformaldehyde for ten minutes, permeabilization is achieved using Triton‐100 (0.2%), and then 5% BSA was added to block the binding of non-specific proteins for 1 h. Afterward, cells were incubated overnight at 4 °C with an anti-γH2AX antibody (ab81299, Abcam, UK), anti-RAD51 antibody (ab88572, Abcam, UK) or anti-cGAS antibody (15102, Cell Signaling Technology, USA). A fluorescent secondary antibody (ab150077, Abcam, UK) was incubated with cells for 1 h on the second day. The fluorescence of γH2AX, RAD51 and cGAS was detected by the ZEISS LSM800.

### Comet assay

A 4 Gy X-ray dose was applied to TE-1 and KYSE-150 cells and samples were collected 24 h after irradiation for DNA damage analysis. The amount of DNA damage has been quantified with the Comet Assay Kit from Trevigen (Gaithersburg, MD) according to the kit’s instructions. Briefly, the cell washing is performed with PBS and resuspended into 5×10^5^ cells/ml. At a temperature of 37 °C, the cells are combined with molten LM-Agarose and pipetted onto a slide. For the purpose of preparing the slides for electrophoresis and DNA precipitation, lysis solution was added, followed by DNA precipitation and SYBR Green I staining. As a result of using Comet Assay IV software (Perceptive Instruments, Bury St. Edmunds, UK), average tail moments of 50 cells per sample were calculated, and the data were presented as means with standard errors from three or more independent experiments.

### Western blot analysis

In this study, TE-1 and KYSE-150 cells are harvested and lysed using RIPA lysis buffer (added with protease inhibitors) in a 4 °C cryogenic centrifuge at 14,000 rpm for 15 min. Using 10% SDS-PAGE, proteins were separated and then transferred to PVDF (polyvinylidene difluoride) membranes (Millipore, MA). A 5% milk blocking solution is used to block the membranes and then kept for overnight incubation with primary antibodies targeting β-Tubulin [AF1216, Beyotime Biotechnology (BB), China], POLI(ab157244, Abcam, UK), RAD51(ab88572, abcam, UK), Ku70/80(ab166822, Abcam, UK), XIAP(2042, Cell Signaling Technology, USA), STING (Cat.13647, Cell Signaling Technology, USA), p-STING (Cat.50907, Cell Signaling Technology, USA), STAT3 (Cat.12640, Cell Signaling Technology, USA), p-STAT3 (Cat.9145, Cell Signaling Technology, USA). As a next step, the membranes are incubated with an anti-rabbit (A0277, BB, China) or anti-mouse secondary antibody (A0286, BB, China) conjugated with horseradish peroxidase. To visualize the bands of protein, enhanced chemiluminescence was used (ECL; BB, China). As an internal control for each sample, the expression of the endogenous β-Tubulin protein is also determined.

### HR and NHEJ assay

In this study, we adopted a novel method capable of measuring both HR and NHEJ activities quantitatively via CRISPR/ Cas9-induced oligodeoxynucleotide (ODN)-mediated DSB repair [34]. Dr. Jiahua Yu from the State Key Laboratory of Radiation Medicine and Protection, School of Radiation Medicine and Protection, graciously gifted this novel system to us. Plating of HEK293T cells is done using 6-well plates with a density of 3×10^5^ cells/plate. Then, a known inhibitor of Mre11-Rad50-Nbs1 complex i.e. mirin is utilized at a concentration of 50 μM to analyze HR activity for its potential suppressive effects whereas NU7441 (1.0 μM), which is a selective inhibitor of DNA-dependent protein kinase, is applied for checking its inhibitory effects on NHEJ activity. 24 h later, plasmids either of ssODN or dsODN are transfected by a reagent Lipofectamine 3000. PCR analysis was then performed for detecting the marker sequence, which serves as a surrogate indicator of HR and NHEJ activity.

### Immunoprecipitation and LC–MS/MS

The cells were cultured in 10-cm dishes, harvested, and lysed for 15 min at 4 °C in RIPA lysis buffer supplied from Beyotime Biotechnology, China. Afterward, the supernatant of centrifuged lysate was incubated with the primary antibody overnight at 4 °C with gentle rocking. The solution was infused with 40 μl/ml of protein A + G agarose (ab193242, Abcam, UK), incubated at 4 °C for 3 h, and then centrifuged. After four times of washing with RIPA lysis buffer, the precipitate was resuspended. Immunoprecipitates and total cell lysates were boiled in 40 μL SDS loading buffer (1×) for 10 min. As a negative control, rabbit IgG (A409, BB, China) for RAD51(ab88572, abcam, UK) while XIAP (2042, Cell Signaling Technology, USA) or mice IgG (A413, BB, China) for POLI were used at 20 μg/mL demonstrating the specificity of this antibody. The protein samples were analyzed by liquid mass spectrometry. The tryptic peptides were dissolved in 0.1% formic acid (solvent A), directly loaded onto a reversed-phase analytical column (15-cm length, 75 μm i.d.). The flow rate was at a constant flow rate of 400 nl/min on an EASY-nLC 1000 UPLC system. The peptides were subjected to NSI source followed by tandem mass spectrometry in Q ExactiveTM Plus (Thermo). The original samples were analyzed by Byonic software (Protein Metrics Inc, USA) to obtain the results of different proteins identified.

### Ubiquitination assay

For ubiquitination assay, cells were incubated with 30 µM MG-132 (TOPSCIENCE, China) for 6 h. Cells were lysed and immunoprecipitated with anti-RAD51 antibody (ab88572, abcam, UK) as described above after incubation. The ubiquitination was detected using an anti-Ub antibody from Santa Cruz Biotechnology, USA(SC166553).

### Statistical analysis

The statistical analysis was carried out with the aid of SPSS 21.0(IBM, USA) and all the graphs were performed using GraphPad Prism 7(GraphPad Software, USA). The results were expressed as the mean values ± SEM from at least 3 independent experiments. Student’s *t* test was used for comparison among different groups, and survival data were analyzed using Kaplan-Meier. Statistical significance was considered at a *P* value < 0.05.

### Reporting summary

Further information on research design is available in the Nature Research Reporting Summary linked to this article.

### Supplementary information

Reporting Summary

## Data Availability

The dataset(s) supporting the findings of this study are included within the article. Requests for materials and supporting data should be addressed to Prof. Zhou jundong.

## Animal studies

This animal study was approved by the Institutional Ethics Committee of Nanjing Medical University (number: KL901200).
